# Reproductive outcomes after TCRS in patients with incomplete and complete uterine septum: a retrospective cohort study

**DOI:** 10.3389/fmed.2025.1555256

**Published:** 2025-04-28

**Authors:** Jian Yang, Cong-Qing Li, Jing-Xian Xia, Wen-Jing Zhu, Jing Wang, Wei He, Qing-Yuan Wang, Wen-Yan Wang

**Affiliations:** ^1^Department of Obstetrics and Gynecology, Second Affiliated Hospital of Anhui Medical University, Hefei, Anhui, China; ^2^Department of Obstetrics and Gynecology, Fu Yang Women and Children’s Hospital, Fuyang, Anhui, China; ^3^Department of Obstetrics and Gynecology, Anhui Women and Children’s Medical Center, Hefei, Anhui, China

**Keywords:** uterine septum, complication, delivery, optimum gestation time, reproductive outcome

## Abstract

**Objective:**

This study aimed to evaluate the effects of transcervical resection of the septum (TCRS) on reproductive outcomes in women of reproductive age with two types of uterine septa.

**Methods:**

In this retrospective cohort study, we evaluated the reproductive outcomes after TCRS in 87 women with an incomplete uterine septum and 35 women with a complete uterine septum. The study was conducted from January 2010 to December 2020 at the Second Affiliated Hospital of Anhui Medical University in China.

**Results:**

In Group A (incomplete uterine septum), TCRS markedly decreased the rates of spontaneous abortion and embryonic arrest (*p* < 0.001) while significantly enhancing the likelihood of term delivery (*p* < 0.001). Similarly, Group B (complete uterine septum) observed a substantial increase in pregnancy rates (from 43.5 to 82.6%), a significant decrease in spontaneous abortions (*p* < 0.05), and an improvement in term delivery rates (*p* < 0.001) post-surgery. The dimension of the septum in cases of Group A did not affect the outcome of adverse reproductive events. However, cesarean section rates were significantly higher in Group B than in Group A (76.5% vs. 47.7%). The optimal time to achieve pregnancy was within the first year for Group A and the 12th month for Group B after TCRS.

**Conclusion:**

In patients undergoing TCRS, a notable decrease in spontaneous abortion rates was observed, alongside an increase in pregnancy and full-term birth rates. However, there was a concomitant rise in cesarean section rates. Furthermore, our findings indicate that patients with an incomplete uterine septum are more likely to conceive earlier than those with a complete uterine septum following surgery, thereby optimizing the timing for pregnancy.

## 1 Introduction

Uterine septum is the most prevalent congenital malformation of the uterus, characterized by a partition that divides the uterine cavity. This partition results from the incomplete fusion of the Müllerian ducts and can vary in extent, leading to either partial or complete division of the uterine cavity and cervical canal, and thus can be classified as incomplete or complete uterine septum (1). The estimated prevalence of this condition ranges from 0.2 to 2.3% among women of reproductive age (2). The majority of women with a uterine septum maintain effective reproductive function (3), while there is substantial evidence linking the uterine septum to miscarriages and preterm deliveries (4). Furthermore, the septum may increase the risk of additional adverse pregnancy outcomes, including fetal malposition, intrauterine growth restriction, placental abruption, and perinatal mortality (2). Given the reduced pregnancy rates and increased miscarriage rates associated with uterine septum, research indicates that surgical resection of the septum substantially enhances pregnancy outcomes (5, 6). Currently, transcervical resection of the septum (TCRS) is the most common surgical method for this condition (3). To the best of our knowledge, no studies have comprehensively assessed post-surgical reproductive outcomes in patients with incomplete uterine septum versus complete uterine septum.

The aim of this study was to compare the reproductive outcomes of pregnant women before and after septum resection and to obtain data on the differences between incomplete and complete uterine septa with respect to various parameters, including the interval between surgeries and subsequent pregnancies, complications, and delivery post-TCRS.

## 2 Methods

**Figure 1:**
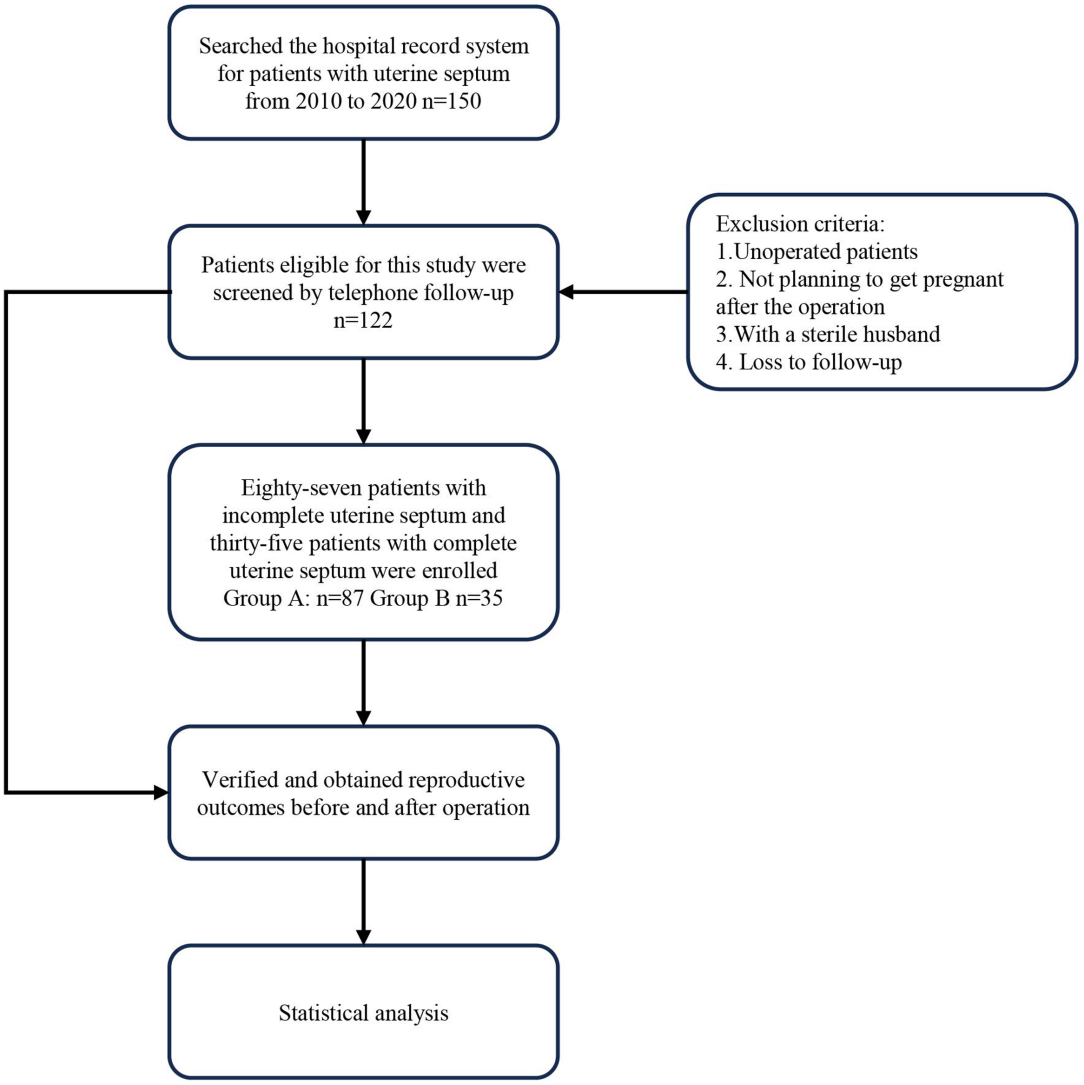
Flowchart of the study.

A total of 150 patients with a uterine septum who attended the Second Affiliated Hospital of Anhui Medical University between January 2010 and December 2020 were enrolled in this study. During the follow-up period, 28 patients were either lost to follow-up or did not meet the inclusion criteria. We followed up with the included patients for 3 years. Among the 122 cases, 87 had an incomplete uterine septum (Group A), and 35 had a complete uterine septum (Group B) (Figure 1). The patients included in this study presented to the clinic with either spontaneous abortion or infertility. All patients underwent an ultrasound examination, during which the uterine septum was discovered, and surgical treatment was recommended.

### 2.1 Surgical equipment and technique

A STORZ (Germany) HD hysteroscope with a 26 Fr diameter resectoscope complete set was used. The dilation medium was 5% mannitol, the dilation pressure was set to 13.4 kPa, and the dilation liquid flow rate was set to 250 mL/min. Monopole electric cutting equipment (needle-like electrode and electric cutting ring, STORZ) was chosen during the operation and used at a power of 70 W. In addition, cervical dilation was required before the insertion of the resectoscope to ensure proper placement and accommodation of the equipment.

#### 2.1.1 Vaginal septum

A vaginal speculum was inserted to gently widen the vaginal opening, enhancing visibility and providing better access for the procedure. The vaginal septum was then carefully excised using a needle-like electrode, allowing for precise cuts while minimizing bleeding. Once the septum was removed, the incisions on the vaginal wall were sutured with absorbable sutures to promote healing.

#### 2.1.2 Incomplete uterine septum

The septum was cut with needle-like electrodes from the caudal end, and the septum was alternately cut horizontally from left to right along the imaginary plane of the bilateral oviduct opening. The electric cutting ring was used alongside the needle-like electrode to cut the septum. Its function is to remove redundant tissue from the anterior and posterior uterine walls to restore a normal uterine cavity shape after the initial incision with the needle-like electrode.

#### 2.1.3 Complete uterine septum

**Figure 2:**
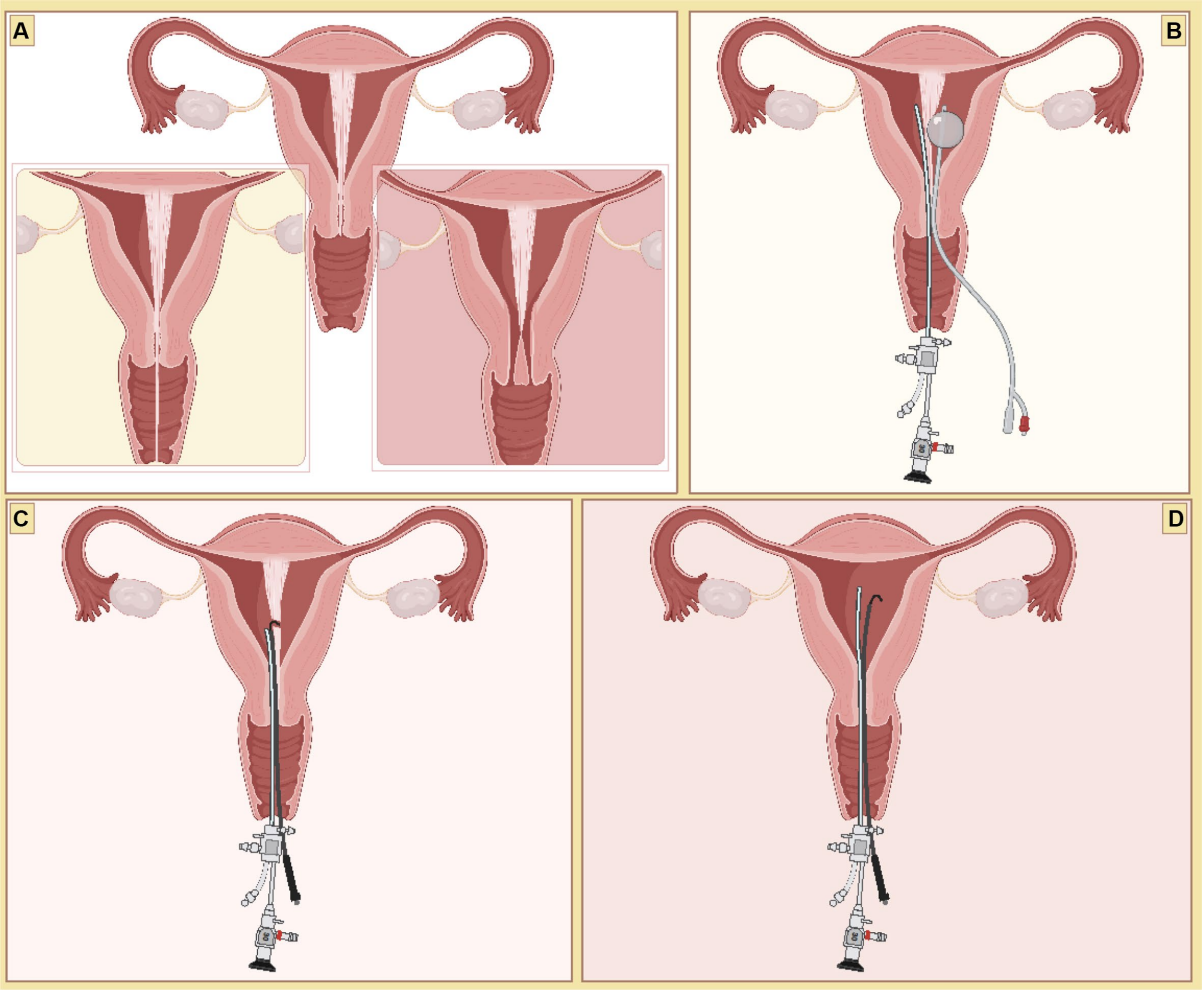
Surgical procedures illustrated in this study. **(A)** A complete uterine septum with a vaginal septum is in the lower left corner, and a double cervix is in the lower right corner. **(B)** Septal weakness was determined through hysteroscopy and catheterization. **(C)** The septum was alternately cut horizontally from left to right along the imaginary plane of the bilateral oviduct opening. **(D)** General view of the uterine cavity after TCRS.

Some septum absorption could be seen above the endocervix, forming a small absence to allow bilateral uterine phase communication. We could use the missing septum as a starting point to cut the septum in the cephalic direction. For a complete uterine septum without septum absence to form bilateral uterine communication, it is difficult to select the site of septum incision. A hysteroscopy was performed on the side with the larger uterine cavity. Then, 3–5 mL of normal saline was repeatedly injected into and aspirated from the contralateral uterine cavity using a Foley balloon catheter, allowing the septum to be observed via hysteroscopy (7–9). After the septum was identified, it was cut into the contralateral uterine cavity at the lower segment of the uterus. The septum was then cut in the same way as for an incomplete uterine septum (Figure 2).

**Figure 3:**
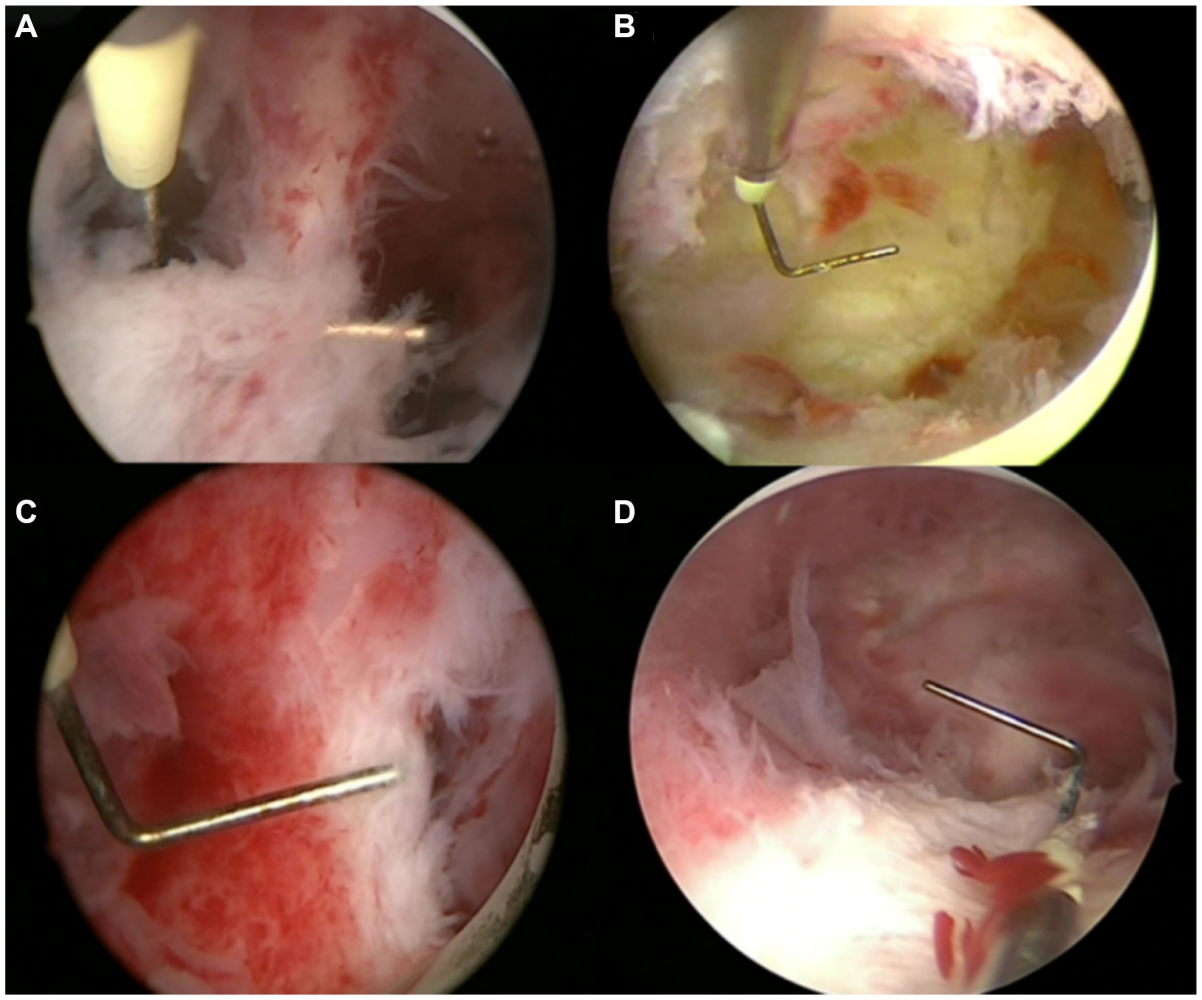
Pictures of the uterine septum before and after surgery. **(A)** Incomplete uterine septum before surgery. **(B)** Incomplete uterine septum after surgery. **(C)** Complete uterine septum before surgery. **(D)** Complete uterine septum after surgery.

Under hysteroscopy, the septum was grayish white and lacked vascular dense tissue, which was the anatomic feature used to determine whether the mediastinum was completely resected. In addition, the fundus muscle thickness was restored to normal uterine wall thickness (Figure 3).

In Group A, two cases of uterine perforation occurred during surgery, and laparoscopic uterine repair was performed. One case of hyponatremia occurred in both Groups A and B, and symptomatic treatment with concentrated sodium was administered during the surgery. Postoperative follow-up revealed one case of mild intrauterine adhesion in Groups A and B. No treatment was required, and both cases resulted in subsequent natural pregnancies.

Statistical analysis was performed using the Wilcoxon rank-sum test for parametric and non-parametric continuous variables. Continuous data are presented as the mean ± standard deviation (SD), and the *χ*2 test or Fisher’s exact test was used to assess categorical variables. A *p*-value of <0.05 was considered statistically significant.

## 3 Results

### 3.1 Basic data

The mean age of the participants in Group A was higher than that of Group B, which was attributed mainly to Group B’s higher infertility rate, which led to shorter timeframes for seeking hospital treatment (28.69 ± 4.32 vs. 26.91 ± 2.90, *p* = 0.028; infertility rates of 9.2% vs. 37.1%, *p* < 0.001). However, the number of pregnancies and infertility cases in the two diseases was significantly different, indicating that the complete uterine septum had a higher probability of infertility (*p* < 0.001). Other comorbidities that could affect the reproductive outcomes of the uterine septum, such as endometriosis and endometrial polyps, were analyzed, and no significant differences were found (*p* > 0.05). A significant proportion of patients with a complete uterine septum also presented with a double cervix and vaginal septum (*p* < 0.0001) (Table 1).

**Table 1:** Basic data of patients with uterine septum.

Target	Incomplete uterine septum	Complete uterine septum	*t(χ^2^)*	p
Patients (*n*)	87	35		
Age (years)	28.69 ± 4.32	26.91 ± 2.90	2.219	0.028
Pregnancy before surgery	74 (85.1%)	18 (51.4%)	15.22	< 0.001
Infertility before surgery	8 (9.2%)	13 (37.1%)	13.68	< 0.001
Comorbidities				
Endometriosis	3 (3.4%)	2 (5.7%)	0.326	0.568
Tubal disease	6 (6.9%)	2 (5.7%)	0.057	0.811
Uterine adhesions	9 (10.3%)	1 (2.9%)	1.86	0.173
Endometrial polyp	4 (4.6%)	2 (5.7%)	0.067	0.795
Vaginal septum and double cervix	2 (2.3%)	16 (45.7%)	37.4	<0.0001

### 3.2 Effect of surgery on reproductive outcomes

Prior to surgery, the pregnancy rate among patients with an incomplete uterine septum was significantly higher than that of those with a complete uterine septum (85.1% vs. 51.4%, *p* < 0.001). However, postoperatively, no significant difference in pregnancy rates was observed between the two groups (85.1% vs. 85.7%, *p* = 0.926). TCRS markedly improved infertility in Group B (from 37.1 to 8.6%).

The number of pregnancies in Group A was almost the same before and after the operation; however, the rates of spontaneous abortion and embryonic arrest were significantly reduced (34.2% vs. 9.8%, *p* < 0.001; 36.7% vs. 4.3%, *p* < 0.001), and the rate of term delivery was significantly improved (75% vs. 6.2%). Although the rate of preterm delivery was increased (3.1% vs. 8.7%), all the fetuses survived during follow-up. There were eight cases of *in vitro* fertilization (IVF) (two cases of fallopian tube disease, three cases of pelvic adhesions, one case of uterine adhesions, and two cases of no complications) (Table 2).

**Table 2:** Comparison of pregnancy characteristics before and after incomplete uterine septum surgery.

	Before surgery	After surgery	*χ* ^2^	*p*
Total pregnancies	161	92		
Ectopic gestation	1 (0.6%)	1 (1.1%)	0.162	0.687
Induced abortion	31 (19.3%)	1 (1.1%)	15.88	<0.0001
Spontaneous abortion	55 (34.2%)	9 (9.8%)	18.41	<0.0001
Embryonic arrest	59 (36.7%)	4 (4.3%)	32.66	<0.0001
Preterm delivery	5 (3.1%)	8 (8.7%)	3.753	0.053
Term delivery	10 (6.2%)	69 (75%)	129	<0.0001

Comparison of pregnancy characteristics before and after complete uterine septum surgery				
	Before surgery	After surgery	*χ* ^2^	*p*
Total pregnancies	28	37		
Ectopic gestation	2 (7.1%)	1 (2.7%)	0.714	0.573
Induced abortion	8 (28.6%)	1 (2.7%)	8.941	0.003
Spontaneous abortion	7 (25%)	2 (5.4%)	5.13	0.024
Embryonic arrest	9 (32.1%)	4 (10.8%)	4.533	0.033
Preterm delivery	1 (3.6%)	1 (2.7%)	0.04	0.841
Term delivery	1 (3.6%)	28 (75.7%)	33.53	<0.0001

The data for Group B showed that surgical resection of the septum could significantly increase the pregnancy rate (51.4% vs. 85.7%), significantly reduce the rates of spontaneous abortion and embryonic arrest (25% vs. 5.4%, *p* < 0.05; 32.1% vs. 10.8%, *p* < 0.05), and increase the rate of term delivery (3.6% vs. 75.7%, *p* < 0.0001). There were five cases of IVF (one case of polycystic ovary syndrome, one case of fallopian tube disease, and three cases with no complications) (Table 2).

### 3.3 Impact of septum size on reproductive outcomes in incomplete uterine septum

Patients with an incomplete uterine septum were divided into two groups during surgery based on whether the length of the septum was greater than half the size of the uterus (2.5–3 cm). Forty-seven women (54%) had a longitudinal septum length larger than one-half of their uteruses, and 40 cases (46%) had a septum size less than one-half of their uteruses. Analysis of spontaneous abortion and embryonic arrest with the main reproductive outcomes found that septum size had no effect on adverse pregnancy outcomes in an incomplete uterine septum (*p* > 0.05) (Table 3).

**Table 3:** Impact of septum size on reproductive outcomes in an incomplete uterine septum.

	*N* = 74	*N* = 87	*χ* ^2^	*p*
Spontaneous abortion	22 (29.7%)	33 (37.9%)	1.196	0.274
Embryonic arrest	30 (40.5%)	29 (33.3%)	0.895	0.344

### 3.4 Complications of pregnancy after operation

During pregnancy and delivery, among all complications, the rates of threatened abortion in early pregnancy among patients with an incomplete uterine septum were similar to those of patients with a complete uterine septum. Notably, nearly half of the women experienced threatened abortion in pregnancies after the operation (47.8% vs. 50%). There was no significant difference in other complications, such as premature rupture of membranes and premature delivery (Table 4).

**Table 4:** Complications of pregnancy after incomplete uterine septum and complete uterine septum surgery.

	Incomplete uterine septum	Complete uterine septum	*χ* ^2^	*p*
Complications (*n*)	46	10		
Threatened abortion	22 (47.8%)	5 (50.0%)	0.016	0.901
Placenta previa	5 (10.9%)	1 (10.0%)	0.233	0.629
Rupture of membranes	4 (8.7%)	1 (10.0%)	0.231	0.631
Preterm delivery	8 (17.4%)	1 (10.0%)	0.01	0.919
Fetal distress	2 (4.3%)	0 (0.0%)	–	1.000^▲^
Hypertension during pregnancy	3 (6.5%)	1 (10.0%)	0.084	0.772
Other complications	2 (4.3%)	1 (10.0%)	0.003	0.956

*▲ Fisher’s exact test.*

### 3.5 Delivery mode after operation

The cesarean section rates after the operation were significantly increased, at 39.7% (27/68) in Group A and 65.4% (17/26) in Group B. In Group A, there were five cases of complications (1 case of hypertensive disease during pregnancy, 3 cases of placenta previa, and 1 case of oligohydramnios), 11 cases of no factors (cesarean section without medical indication), and 41 cases of vaginal delivery. In Group B, there were 1 case of complications (hypertensive disease during pregnancy), 10 cases of no factors, and 9 cases of vaginal delivery. Among the reasons for the high cesarean section rate, no factors were the main factors among patients with a complete uterine septum (*p* = 0.02). Patients with an incomplete uterine septum were statistically more likely to have a vaginal delivery than those with a complete uterine septum (*p* = 0.026) (Table 5).

**Table 5:** Causes of cesarean section after surgery in the incomplete uterine septum and complete uterine septum groups.

	Incomplete uterine septum	complete uterine septum	χ^2^	*p*
*N*	68	26		
History of cesarean section	3 (4.4%)	0 (0.0%)	–	0.558^▲^
Abnormal fetal position	4 (5.9%)	2 (6.9%)	0.023	0.880
Macrosomia	2 (2.9%)	3 (10.3%)	1.107	0.313
Fetal distress	2 (2.9%)	1 (3.45%)	0.187	0.665
Complications	5 (7.4%)	1 (3.45%)	0.023	0.880
No factors	11 (16.2%)	10 (41.4%)	5.384	0.020
Vaginal delivery	41 (60.1%)	9 (34.5%)	4.981	0.026

*▲ Fisher’s exact test.*

### 3.6 Best time for pregnancy after operation

The pregnancy rate within 2 years after the operation was 94.1% (64/68) in Group A after the operation. There were 26 pregnancies in Group B after the operation, and the pregnancy rate within 2 years was 92.3% (24/26). The majority of the first pregnancies occurred in the first year after the operation, accounting for 80.9% (55/68) of the total number of pregnancies in Group A. The majority of the first pregnancies occurred between the 6th and 24th month after the operation, accounting for 69.2% (18/26) of all pregnancies in Group B. The duration of pregnancy among patients with an incomplete uterine septum was significantly lower than that among patients with a complete uterine septum (*Z* = −2.286, *p* = 0.022) (Table 6).

**Table 6:** Number of pregnancies over time (months) after surgery in patients with an incomplete uterine septum and a complete uterine septum.

	Incomplete uterine septum	Complete uterine septum
0–3 months	18	2
3–6 months	14	4
6–12 months	23	11
12–24 months	9	7
>24 months	4	2

The duration of pregnancy in an incomplete uterine septum was significantly lower than that in a complete uterine septum. Analysis using the Wilcoxon rank-sum test showed a significant difference between the two groups (*Z* = −2.286, *p* = 0.022).

## 4 Discussion

Currently, there is variability in the necessity for surgical intervention among patients diagnosed with a uterine septum. In a cohort study conducted in 2020, it was observed that septal hysterectomy did not yield an increase in live birth rates, nor did it decrease the incidence of pregnancy loss or preterm birth compared to expectant management (10). This finding was subsequently confirmed by an international multicenter open-label randomized controlled trial in 2021, although the study was limited by a small sample size and a prolonged recruitment period (11). Conversely, Frank was the first to propose the feasibility of transcervical resection of the septum (TCRS) in 1981 (12). Presently, various methodologies are utilized to address the uterine septum, encompassing hysteroscopic cold scissor incisions, unipolar or bipolar incisions, and laser incisions. Nicola Colacurci’s research (13) revealed no significant differences in reproductive outcomes, such as premature birth rates or spontaneous abortion rates, between bipolar and unipolar electrode surgeries. A network meta-analysis (14) indicated that hysteroscopic scissors were associated with higher pregnancy rates than the resectoscope. Moreover, Berta Esteban Manchado’s investigation (15) suggested that laser surgery, boasting a 78.9% pregnancy rate, is a viable and safe alternative, demonstrating sufficient efficacy in reproductive outcomes to merit further inquiry. Hysteroscopic uterine septum resection may yield positive effects on enhancing reproductive outcomes in patients with recurrent spontaneous abortion or infertility (16); notably, women with a complete uterine septum may experience a relatively significant increase in postoperative pregnancy rates (17). This study established that TCRS significantly diminishes the incidence of spontaneous abortion and embryonic arrest while also enhancing full-term delivery rates.

TCRS is a well-established treatment for septate uteri in patients experiencing infertility or spontaneous abortion. Although hysteroscopic surgery is highly effective, there is a small risk of serious complications during and after the procedure, which may threaten patient safety (18, 19). Among these, uterine perforation and hyponatremia are particularly critical and necessitate careful preventive measures. Conventional TCRS techniques typically utilize a Foley catheter for septum localization and rely heavily on the surgeon’s expertise to excise excess tissue and restore normal uterine cavity morphology. In comparison, ultrasound-guided septal resection presents notable advantages (20); (1) real-time ultrasound imaging minimizes the risk of uterine perforation by preventing excessive tissue removal; (2) enhanced visualization ensures precise anatomical guidance, thereby reducing operative time and lowering the incidence of complications such as hyponatremia and residual septum. Consequently, for the complex uterine septum, the integration of ultrasound guidance into TCRS holds significant potential for improving both the safety and efficacy of the procedure.

We investigated the effect of septal size on pregnancy outcomes in women with an incomplete uterine septum and found no significant difference in the rates of spontaneous abortion and embryonic arrest, regardless of septal size, with both high and low incidences observed. Similarly, Paradisi R (21) reported that there was no difference between preoperative spontaneous abortion and infertility rates, irrespective of septal size, and that hysteroscopic septoplasty significantly improved reproductive performance.

The results do not support elective hysteroscopic incision of the septum in asymptomatic patients or before the first pregnancy, including cases of breech presentation, gestational hypertension, preterm birth, and growth retardation (22). However, this study concluded that the rate of threatened abortion (characterized by abdominal pain and a small amount of vaginal bleeding) in early pregnancy significantly increases after the operation in patients with a uterine septum. Nevertheless, with active treatment, the pregnancy can continue, with the majority of patients delivering at full term and only a few delivering preterm. The high rate of threatened abortion may be attributed to the short repair time for the endometrial injury caused by the operation, the failure to restore normal uterine function, and zygote implantation in the septum. It is not clear whether appropriately prolonging the pregnancy duration after the operation would reduce the risk of threatened abortion, so further data collection is necessary to clarify this issue. Placenta previa in patients with an incomplete uterine septum operation is related mainly to the high rate of spontaneous abortion and embryonic arrest, which are caused by multiple uterine operations. If the uterine septum is found by ultrasound because of multiple spontaneous abortions or embryonic arrest, the septum can be actively treated to prevent an increase in pregnancy complications such as placental abnormalities and threatened abortion. No significant association was found between other comorbidities and whether the uterine septum was operated on.

After TCRS, an increase in cesarean section rates has been observed; however, the mechanisms by which septum resection contributes to this increase remain unclear. Fox NS (23) reported that hysteroscopic resection of the uterine septum is linked to a higher incidence of cesarean delivery in nulliparous women with viable pregnancies. This study identified factors such as abnormal fetal position, placenta previa, and fetal distress, which contribute to elevated cesarean section rates postoperatively in cases of incomplete uterine septum. These factors may similarly affect patients with a complete uterine septum. No factors affecting the rate of cesarean section (cesarean section without a medical indication) were significantly higher. Patients with a complete uterine septum tend to have a lower pregnancy rate before surgery and a longer pregnancy interval after surgery. Additionally, the presence of the vaginal septum and double cervix may increase the psychological stress of pregnant women, leading to a preference for cesarean section. Furthermore, excessive resection of the septum may lead to a weaker uterine wall, which could stretch further during pregnancy and increase the risk of uterine rupture (24). In addition, differences in the histology of the septum and myometrium may disrupt uterine polarity during labor, leading to uterine weakness, excessive uterine contractions, and increased risk of postpartum bleeding or uterine rupture. To avoid related complications during vaginal delivery, which may lead to poor pregnancy outcomes, cesarean section was chosen. Although the complications can be quite serious, the probability of their occurrence appears to be low. Some investigators (25) reported that there was no case of uterine rupture after the operation. In addition, Vid Jansa’s study showed that only four cases were found to have uterine rupture during cesarean section delivery (26). Therefore, the high rate of cesarean section in patients with a complete uterine septum is primarily due to psychological factors. By addressing the fears of pregnant women early on and closely monitoring maternal and fetal safety during labor, unnecessary cesarean sections can be prevented.

After TCRS, endometrial wound healing reaches 100% within 2 months (27), but no study has calculated the optimal time for postoperative natural pregnancy. Panagiotis Bakas (28) reported that the highest pregnancy rate was within 15 months after surgery. A retrospective cohort study by Murat Berkkanoglu (29) found no differences in the pregnancy or miscarriage rates of 282 women who underwent IVF/ICSI at <9, 10–16, or >17 weeks after surgery. During the follow-up of our study, the pregnancy rate of the patients with an incomplete uterine septum was highest within the first year after surgery, while that of the patients with a complete uterine septum was highest at the 12th month after surgery. The longer pregnancy interval in this study may be attributed to poor preoperative pregnancy outcomes, with patients delaying sexual intercourse due to concerns about the extent of surgical trauma and the need for an extended recovery period for complete uterine septum repair. In addition, a lack of detailed knowledge about the septum’s width or its depth into the myometrium, along with insufficient understanding of the postoperative recovery of deep tissues, may contribute to a longer interval between surgery and subsequent pregnancy. There are still adverse pregnancy outcomes, such as abortion and embryonic arrest after surgery. Due to the lack of data regarding the precise timing of abortion following TCRS, this study did not explore the correlation between abortion rates and the postoperative pregnancy interval, aiming to prevent unintended pregnancies resulting in spontaneous abortion or embryonic arrest.

The potential limitations of our study include the relatively small sample size of patients with a uterine septum, the inability to completely exclude other confounding infertility factors, and the retrospective nature of the study, which may cause some bias. This study provided clinically useful data to assist and clarify the clinical diagnosis and determine treatment. We plan to conduct a randomized controlled trial in the future to objectively understand the impact of TCRS on reproductive outcomes. More data will be added in the future.

## 5 Conclusion

In this study, we found that in patients who underwent TCRS, the rate of spontaneous abortion decreased, and the rates of pregnancy and full-term birth increased; however, the rates of cesarean section increased, but these complications are acceptable pregnancy outcomes because they do not result in a high miscarriage rate and can better improve the pregnancy rate and full-term birth rate. We advocate for careful timing of pregnancy to mitigate the risk of unintended pregnancies leading to adverse outcomes post-operation. Specifically, in our study, the pregnancy rate in patients with an incomplete uterine septum was higher within the first year after surgery, while the pregnancy rate for patients with a complete uterine septum was highest at approximately 12 months post-operation. We have suggested this conclusion only tentatively, with the understanding that further updates of future results will help validate the ideal timing for pregnancy after surgery.

## Data Availability

The raw data supporting the conclusions of this article will be made available by the authors, without undue reservation.
